# Extragenital Bullous Lichen Sclerosus in a Male Pediatric Patient and Dermoscopic Keys: A Case Report and Literature Review

**DOI:** 10.7759/cureus.99957

**Published:** 2025-12-23

**Authors:** Stephanie Y Zhang, Natalie Garcia, Lindy S Ross, Audrey Chan

**Affiliations:** 1 Department of Dermatology, Baylor College of Medicine, Houston, USA; 2 Department of Dermatology, University of Texas Medical Branch, Galveston, USA

**Keywords:** bullous, clobetasol propionate, extragenital, hemorrhagic lesions, lichen sclerosus et atrophicus, non-genital, pediatric dermatology

## Abstract

We describe the case of a 12-year-old male who presented with a one-and-a-half-month history of an asymptomatic, rapidly expanding erythematous hyperkeratotic plaque on the left shoulder. On clinical exam, the patient was also found to have an ivory white atrophic plaque on the left clavicle. Given the classic lichen sclerosus (LS) lesion on the patient’s clavicle, the lesion in question on his left shoulder was diagnosed as extragenital bullous LS (EBLS). The bullous variant of LS, while common in adult literature, is rarely reported in the pediatric population. The patient had a moderate clinical response to a high-potency topical corticosteroid. A review of this case, along with the literature, describes the epidemiology, clinical features, pathophysiology, and available treatment options for EBLS in the pediatric population.

## Introduction

Lichen sclerosus (LS) is a chronic inflammatory skin disease with a largely unknown pathogenesis that most commonly affects the anogenital areas. While the disease can occur at all ages and in both sexes, it has been estimated to affect prepubertal and postmenopausal women compared to men in a 3:1 ratio [1]. While the pathogenesis of LS is still largely unknown, current literature suggests autoimmune factors play a role, specifically autoantibodies to extracellular matrix protein 1, a genetic predisposition with HLA-DQ7, and association with other autoimmune diseases [2-3].

Classically, LS presents as porcelain-white atrophic plaques. Approximately 85% of LS are genital cases, and of the extragenital cases, only 6% present as isolated extragenital lesions [4]. Extragenital LS typically affects the back, chest, and breasts [5]. A bullous variant has been recognized in adult literature and is described as hemorrhagic blisters in the setting of LS [6]. This variant has been rarely reported in children, with only one case report to date on extragenital bullous LS (EBLS) in a 14-year-old girl [7]. We present another case of pediatric EBLS in a 12-year-old boy.

## Case presentation

A 12-year-old male with no significant past medical history was referred to the dermatology clinic after presenting to his pediatrician with a one-and-a-half-month history of an asymptomatic erythematous hyperkeratotic plaque on his left shoulder. The patient reported that the lesion began as a small dry patch but became progressively more red and raised. There was no associated pain, bleeding, pruritus, or history of previous trauma.

**Figure 1:**
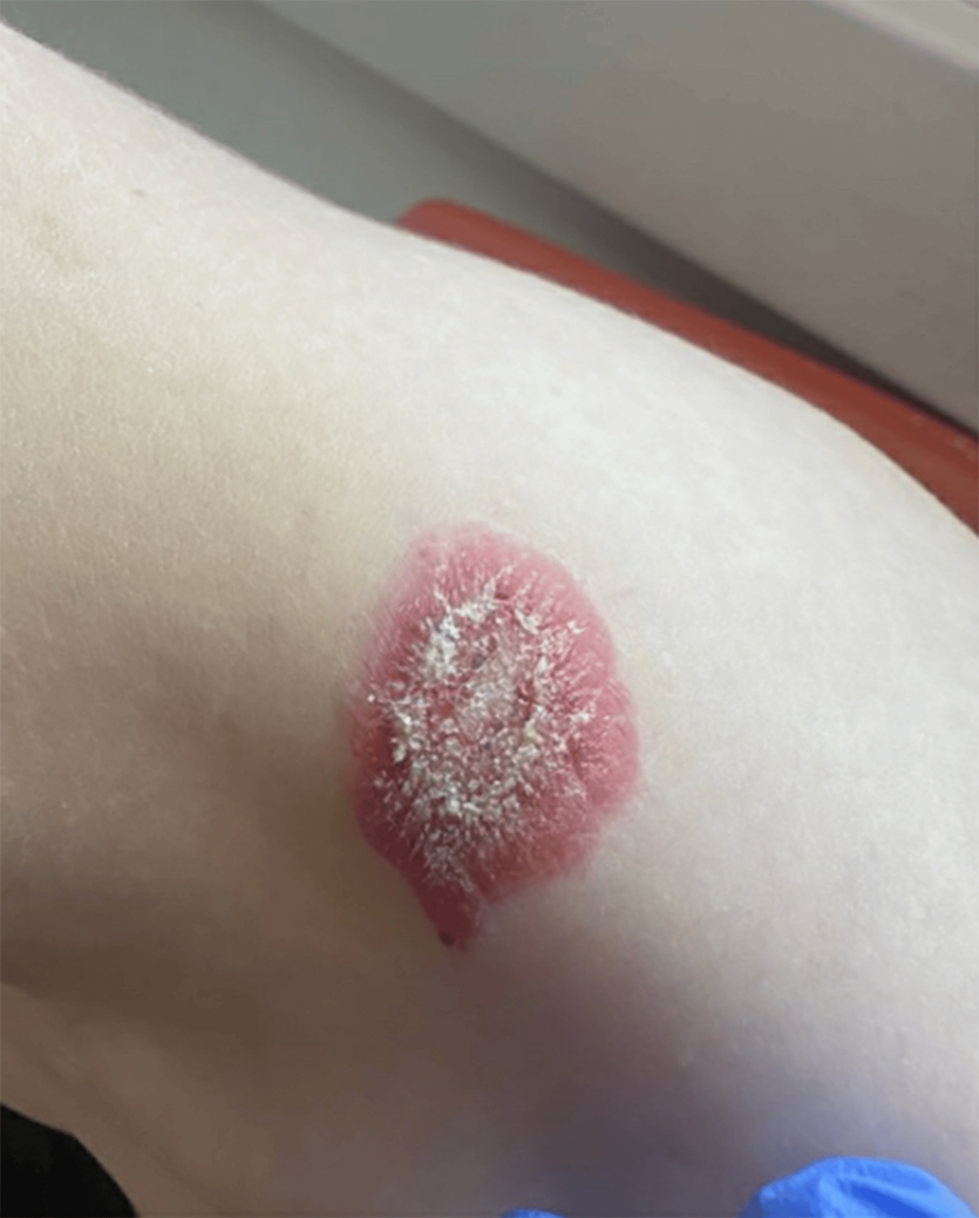
3 × 2 cm, erythematous, hyperkeratotic plaque located on left shoulder

**Figure 2:**
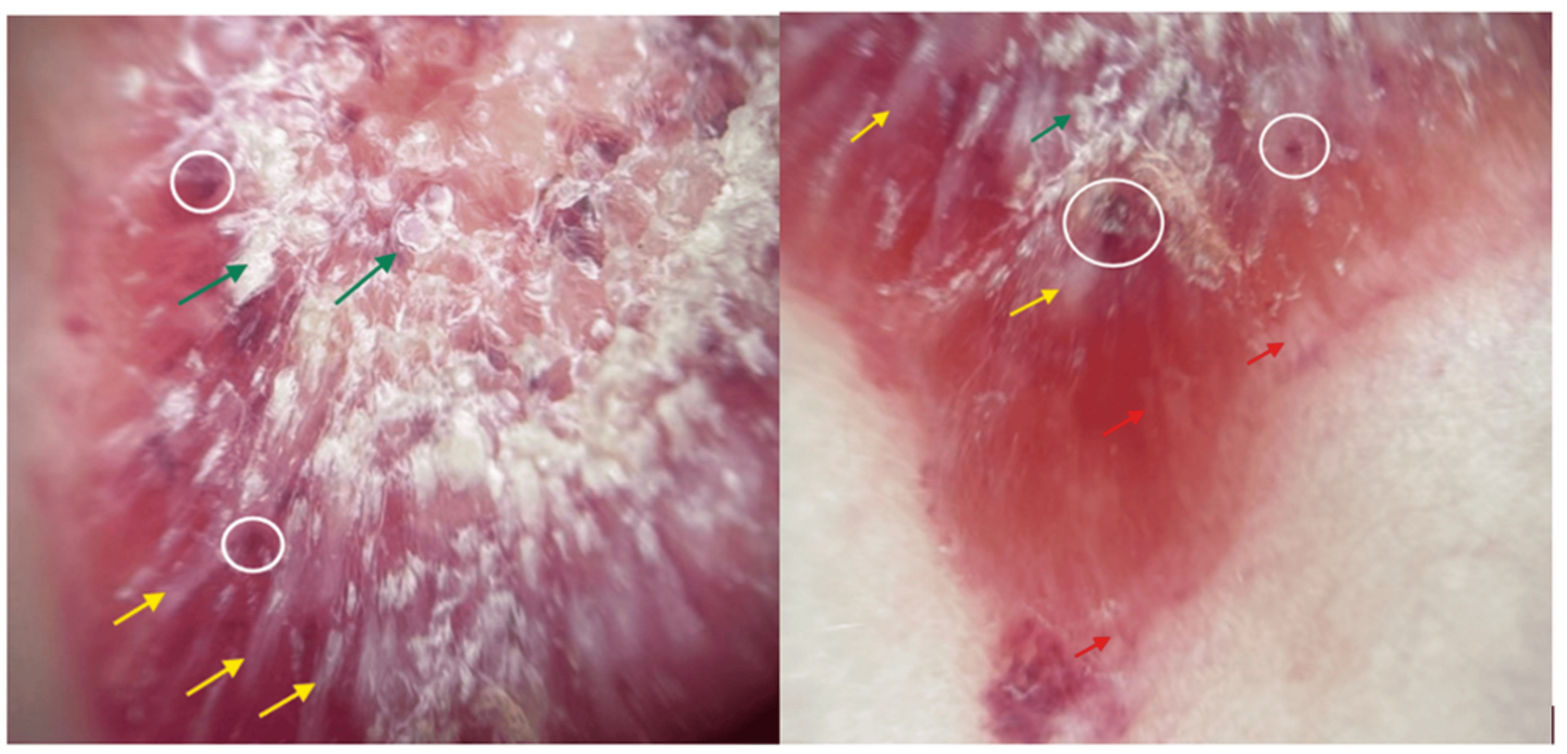
Non-polarized dermoscopy of the lesion Green arrow - marked central hyperkeratosis and keratin plugging; yellow area - peripheral white structureless areas and linear white streaks; red arrow, right image - rare linear vessels at peripheral edge; white circles - numerous vascular lacunae and milky red areas.

**Figure 3:**
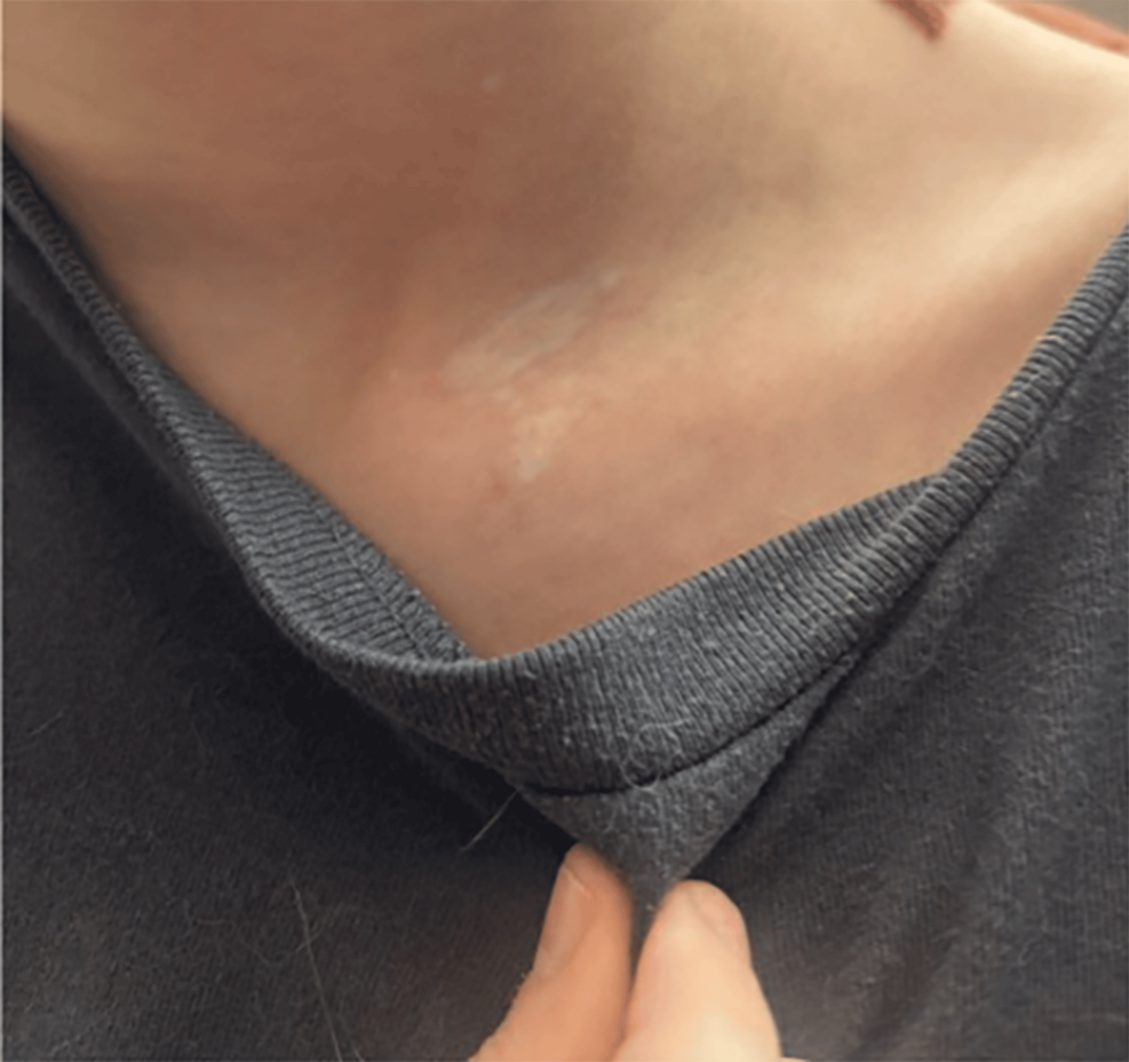
Hypopigmented scaly patch and scattered hypopigmented macules on the left medial clavicle

**Figure 4:**
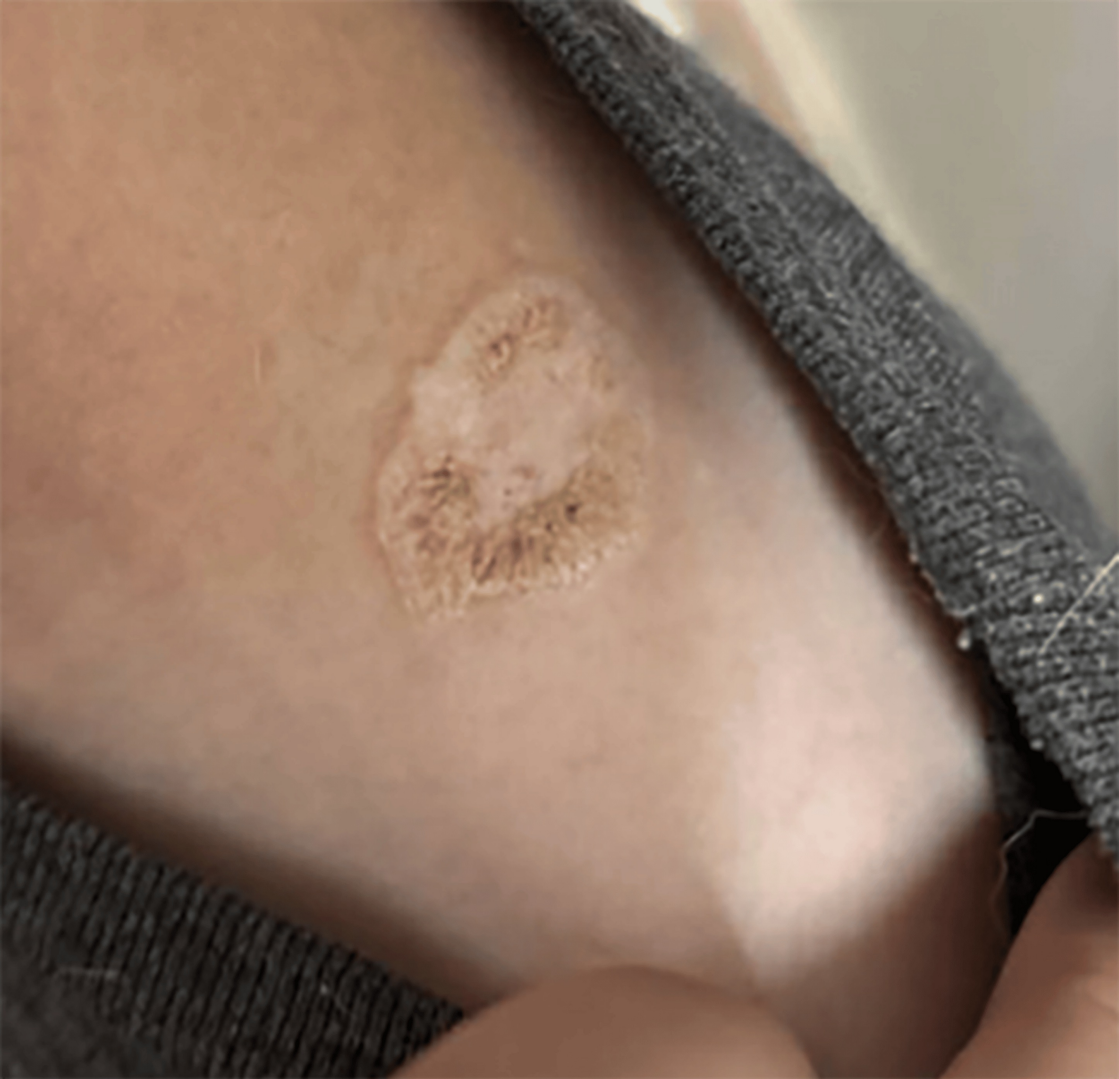
Resolution of hemorrhage and significant improvement in overlying bullae with eight weeks of clobetasol 0.05% ointment

On initial review of photos at the request of the pediatrician, our initial differential diagnoses favored inflammatory or infectious processes such as psoriasis, nummular eczema, or tinea. The in-person exam was notable for a 3 cm × 2 cm raised, well-demarcated, erythematous, hyperkeratotic plaque on the patient’s left shoulder (Figure 1). Dermoscopy was notable for red globules, bright white structures, and scale (Figure 2). In addition, the patient was found to have an ivory white atrophic plaque on the left medial clavicle and scattered hypopigmented macules on the neck consistent with extragenital LS (Figure 3). The anogenital area was clear. Thus, a clinical diagnosis of extragenital LS with a hemorrhagic bullous component was made. The patient was initiated on 0.05% clobetasol ointment twice daily with marked improvement in the plaque at follow-up (Figure 4).

## Discussion

Bullous LS is a rare variant of LS that is thought to occur due to extensive vacuolar degeneration of the epidermal basal cell layer, resulting in subsequent fragility of the dermal-epidermal junction and edema in the papillary dermis [8]. Bullae are hypothesized to be a transient component of early lesions that heal before the classic ivory white atrophic plaques of LS appear, and bullous LS lesions are more resistant to treatment [9]. Hemorrhage is often referenced in conjunction with bullae, likely due to the disruption and loss of collagen support of the dermal capillaries that results in subsequent hemorrhage within the bullae [10]. Classic LS typically presents with symptoms of dryness and pruritus; however, these features are less common in bullous LS, as observed with our patient, who reported no associated symptoms with the lesion [11].

Dermoscopically, the essential components of EBLS are described as white structureless areas, follicular plugs, white chrysalis-like structures, and variable vascular patterns [12]. Histologically, the structureless white areas visualized on dermoscopy represent hyperkeratosis and epidermal atrophy, and the linear white streaks correspond to homogenization of collagen in the superficial dermis and sclerosis (better visualized with polarizer) [13]. Early and late phases of lesions may also demonstrate peripheral linear vessels, which represent telangiectasias or dilated blood vessels, as seen with our patient (Figure 2, right image) [13]. Hemorrhagic areas might also be visualized with darker shades representing older hemorrhage [12]. The red lacunae seen in our case likely correspond to areas of hemorrhage in the superficial dermis and stratum corneum.

While etiology remains unknown, there is evidence suggesting a potential association with genetic susceptibility and autoimmune mechanisms [2,14]. A genetic predisposition to LS is associated with HLA-DQ7, and 80% of patients with LS have IgG autoantibodies to extracellular matrix protein 1 (ECM1). Reports also associate LS with other autoimmune conditions such as thyroid disease, pernicious anemia, type 1 diabetes, and alopecia areata [7]. The Koebner phenomenon is also well established as an etiology of LS, where typical lesions develop in pre-existing scars or areas associated with repeated trauma [14].

A review of literature resulted in 35 reports of Japanese origin, in which bullous LS was observed in solely extragenital regions, suggesting that blister formation is associated with extragenital locations [15]. Of the 32 cases of exclusive EBLS that have been reported in English literature, all cases were reported in older adults, apart from a prior report of EBLS in a 14-year-old girl [7, 11]. This patient also reported an EBLS lesion on the neck, which was diagnosed and treated as tinea before biopsy diagnosis in the dermatology clinic. Both this case and our patient’s case highlight the degree of scale/hyperkeratosis that can be seen with extragenital bullous LS mimicking more common pediatric diagnoses such as tinea, eczema, or psoriasis.

A recent retrospective review of 55 patients with extragenital LS found that 67.7% of EBLS patients were later diagnosed with genital LS, and only 57% of those later diagnosed with genital LS endorsed genital symptoms [16]. These findings are an important reminder that clinicians remain vigilant for genital involvement at the time of diagnosis and during ongoing follow-up, even in patients who deny symptoms. Hemorrhage in the setting of genital LSA has been mistaken as a sign of child abuse, so documentation of genital LS can also help prevent undue alarm from other evaluating providers.

Current recommendations for the treatment of EBLS are based on prior case reports, given the rarity of this condition. Topical corticosteroids remain the first-line therapy for all forms of LS, with the majority of EBLS cases treated with topical clobetasol propionate 0.05% once or twice daily, however, often with reports of less clinical response than classic LS [11]. Specific case reports of EBLS have confirmed that while topical clobetasol resolves the blistering and hemorrhage as seen in our patient at their follow-up (Figure 4), it had little effect on the underlying atrophic plaque [10,14,17]. Additional therapies reported in the literature include topical calcineurin inhibitors, surgical excision, and phototherapy [7,18].

## Conclusions

This case highlights a clinical reminder of extragenital bullous LS in pediatric patients. Although rare in children, to our knowledge, we report the second reported case of EBLS in a pediatric patient. Both pediatric patients presented with lesions on or near the neck. Our case highlights the prominent hyperkeratosis that can be seen with EBLS mimicking infectious or papulosquamous processes, especially in the era of telemedicine. Recent studies highlight the need for a thorough genital exam even in asymptomatic patients, as most patients with EBLS either present with genital LS concomitantly or can develop genital LS in the future, with risk for scarring or squamous cell carcinoma if untreated.
